# Membrane Ballooning in Aggregated Platelets is Synchronised and Mediates a Surge in Microvesiculation

**DOI:** 10.1038/s41598-017-02933-4

**Published:** 2017-06-05

**Authors:** Ejaife O. Agbani, Christopher M. Williams, Ingeborg Hers, Alastair W. Poole

**Affiliations:** School of Physiology, Pharmacology and Neuroscience, University of Bristol, Medical Sciences Building, Bristol, BS8 1TD United Kingdom

## Abstract

Human platelet transformation into balloons is part of the haemostatic response and thrombus architecture. Here we reveal that in aggregates of platelets in plasma, ballooning in multiple platelets occurs in a synchronised manner. This suggests a mechanism of coordination between cells, previously unrecognised. We aimed to understand this mechanism, and how it may contribute to thrombus development. Using spinning-disc confocal microscopy we visualised membrane ballooning in human platelet aggregates adherent to collagen-coated surfaces. Within an aggregate, multiple platelets undergo ballooning in a synchronised fashion, dependent upon extracellular calcium, in a manner that followed peak cytosolic calcium levels in the aggregate. Synchrony was observed in platelets within but not between aggregates, suggesting a level of intra-thrombus communication. Blocking phosphatidylserine, inhibiting thrombin or blocking PAR1 receptor, largely prevented synchrony without blocking ballooning itself. In contrast, inhibition of connexins, P2Y_12_, P2Y_1_ or thromboxane formation had no effect on synchrony or ballooning. Importantly, synchronised ballooning was closely followed by a surge in microvesicle formation, which was absent when synchrony was blocked. Our data demonstrate that the mechanism underlying synchronised membrane ballooning requires thrombin generation acting effectively in a positive feedback loop, mediating a subsequent surge in procoagulant activity and microvesicle release.

## Introduction

Platelets are critical mediators of the haemostatic response. In addition to forming the classical platelet aggregate, platelets also support coagulation by exposing phosphatidylserine (PS) on their plasma membrane resulting in the binding and activation of the prothrombinase complex and thrombin formation. Thrombin is a major activator of platelets in response to vascular injury. Genetic disruption or deletion of the thrombin receptor or a pharmacological inhibition of thrombin formation significantly impairs platelet recruitment to wound sites and results in bleeding diathesis^1–4^. While most of the key players in the haemostatic response have been identified, a dearth of knowledge still exists in the current understanding of how several events such as PS exposure and the generation of soluble diffusible agonists for platelet activation are spatiotemporally and optimally coordinated to arrest bleeding and stimulate the healing of injured vessels^5^. Furthermore, the morphological transformation into balloons that platelets undergo at wound sites have long been identified^6, 7^; indeed, platelets have also been reported to form balloon-like structures in suspension in response to thrombin stimulation^8–10^, *in vitro* on collagen-coated surfaces^11–13^, and in *in vivo* injury models^14–16^. However, only recently has membrane ballooning been recognized as a pivotal and targetable mechanism in the platelet procoagulant response^17^. The ballooned platelet membrane as well as the platelet body (also referred to as ‘cap’) can become PS positive and thereby contribute to thrombin generation^8, 17^. Pharmacological intervention that blocked platelet ballooning and associated procoagulant membrane spreading profoundly reduced local thrombin generation and *in vivo* thrombus formation, emphasising the importance of this process in haemostasis^17^.

Membrane ballooning has also been suggested as a novel mechanism for the generation of microvesicles^17^. These are membrane fragments, submicron in size and are released from activated blood cells. However, by far the most abundant type of microvesicles found in the human circulation are platelet-derived and express CD62P, CD41, CD42 and CD61^18–20^. The role of microvesicles in thrombosis and haemostasis is linked to their ability to amplify thrombin generation, even in platelet poor plasma^21^. We recently showed that individual platelets adherent to a collagen-coated surface can form extensive PS positive spread membrane structures that break up into procoagulant microvesicles, thereby massively amplifying the procoagulant response^17^. Clinical conditions highlighting this procoagulant role include Scott’s syndrome and Castaman’s disease in which platelets show impaired ability to generate microvesicles and patients suffer a bleeding defect^22–24^. Furthermore several bodies of evidence also point to a role for microvesicles in the pathogenesis of coronary artery diseases and stroke^25–29^. While, the cellular mechanisms underlying the formation of circulating microvesicles is well characterised^30^, the spatio-temporal dynamics of their release at wound sites remain largely unknown. Furthermore, at present little is known about the dynamics of platelet ballooning during aggregate or thrombus formation and how this temporally contributes to thrombin and microvesicles formation.

In this study, we therefore aimed to elucidate the spatiotemporal regulation of PS exposure, thrombin generation and microvesicle release by studying membrane ballooning in platelet aggregates formed in plasma on a collagen-coated surface using four-dimensional live-cell imaging approach. In observing ballooning in platelet aggregates, rather than single cells^17^, we noted that multiple platelets underwent ballooning in a synchronised manner, leading to a surge in the release of microvesicles. The mechanism underlying the synchrony of ballooning is the result of thrombin generation, at the core of the aggregate, where platelets directly in contact with collagen balloon and account for the initial central PS exposure within growing aggregates. Potentially, this may in *in vivo* setting form a novel positive feedback activation, leading to synchronised ballooning of platelets in peripheral areas of the aggregate and the generation of procoagulant microvesicles for the rapid amplification of thrombin generation at wound sites.

## Results

### Ballooning and procoagulant-spreading in platelets within platelet-rich plasma

**Figure 1:**
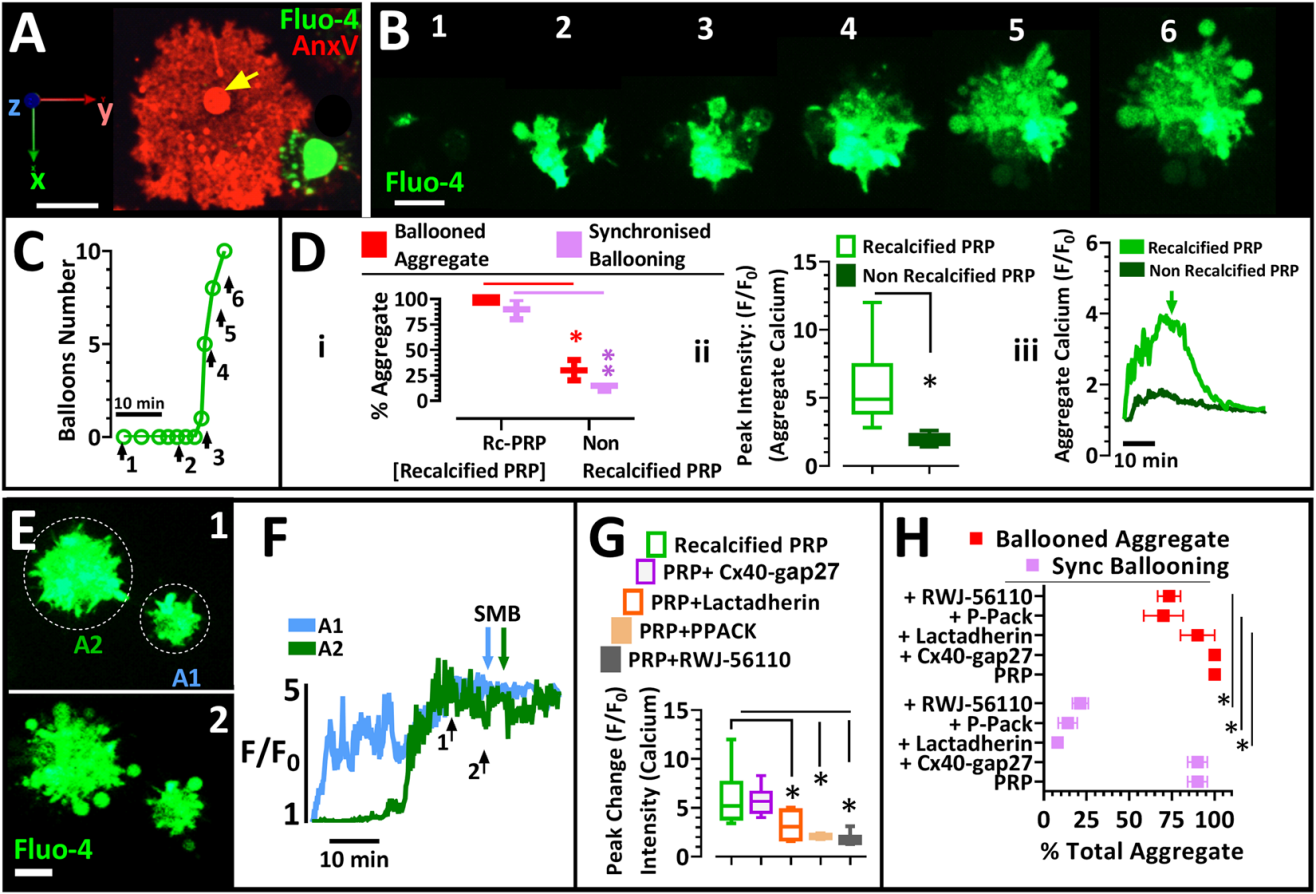
Human platelet aggregation in platelet-rich-plasma is characterised by synchronous membrane ballooning and is extracellular calcium-dependent. (**A**) Platelets in re-calcified platelet-rich plasma (Rc-PRP) were labelled with the calcium indicator Fluo-4 and allowed to adhere to collagen-coated surface. Superimposed extended focus images of Alexa568-Annexin-V (AnxV, red) and Fluo-4 (green) show the presence of a ballooned and procoagulant-spread (BAPS) platelet (red), yellow arrow points to the ballooned part of this platelet. (**B**) Platelets in Rc-PRP labelled with Fluo-4, undergo synchronised membrane ballooning (SMB) as they aggregate on collagen matrix. Numbers relate to arrows in C. Related to Movie S1 (**C**) Number of balloons formed over time in representative aggregate from B. Numbered arrows correspond to numbered images in B. (**D**) The absence of extracellular Ca^2+^ (with non-recalcification) blocks synchronous membrane ballooning. D-i, percentage of aggregates which showed SMB relative to aggregates with ballooned platelets at 1 hr. D-ii, peak Ca^2+^ intensity (F/F_0_) of aggregates. D-iii, calcium fluxes of representative aggregates with time. Green arrow indicates point of SMB in re-calcified PRP. (**E**) Aggregate formation of Fluo-4-labelled platelets in Rc-PRP. Images show platelet aggregations at time points corresponding to numbered arrows in (**F**), which shows calcium fluxes of aggregate numbers A1 and A2. Coloured arrows indicate onset of SMB. (**G**&**H**) Platelets in Rc-PRP were labelled with Fluo-4 and allowed to adhere to a collagen-coated surface for 1 hr. (**G**) Peak change in cytosolic calcium is shown, in absence or presence of connexin40 inhibitor (Cx40-GAP27; 100 g/ml), 2% ^v^/_v_ lactadherin, 100 µM PPACK or 50 µM RWJ-56110. (**H**) Percentage of total aggregates, showing SMB or total ballooning (i.e. synchronised plus non-synchronised). Charts in Di, Dii, G and H show interleaved box plots with whiskers showing minimum to maximum values, median, and interquartile range. Data analysis was by Wilcoxon Signed Rank Test (D-i) or Friedman test, followed by Dunn’s multiple comparison test (G,H). *P < 0.05 was considered significant. Scale bars in A, B and E represent 5μm. Data were from 10–15 aggregates (of 7–40 platelets) per view; and 35 ± 5 aggregates were analysed for each of 5 donor blood samples.

The adhesion of individual washed platelets to a collagen-coated surface results in the formation of ballooned and procoagulant-spread (BAPS) platelet phenotype, which massively contribute to localised thrombin generation^17^. Here we confirm that ballooning and procoagulant-spreading also occurs in platelets within platelets-rich plasma (PRP) on a collagen-coated surface and that a single BAPS platelet (Fig. 1A) covers a wide surface area, equivalent to the size of a platelet aggregate (Fig. 1B). To explore the role and regulation of balloon formation in a growing aggregate, we studied their formation in PRP on a collagen-coated surface using 4D live-cell fluorescence imaging of fluo-4-loaded platelets.

### The calcium dependence of synchronised membrane ballooning

We observed intracellular calcium [Ca^2+^]_i_ spikes in individual platelets within the growing aggregate (Fig. 1B and Movie S1), which showed non-synchronous spiking until just prior to ballooning, where spiking ceased and intracellular [Ca^2+^]_i_ then remained in each platelet at a high, plateau level as though synchronised. Importantly, the platelets on the outer layer of the aggregate formed balloons in a synchronised manner (images 4–6, Fig. 1B,C and Movie S1). Platelets were judged to aggregate when cell-cell contact was achieved and sustained throughout the period of visualisation. Membrane ballooning in aggregating platelets was deemed to have been synchronised if preceded by a synchronisation of the calcium fluxes in the aggregating platelets and when ≥60% of balloons formed by these platelets appeared within a 5 minute period. Synchronised ballooning was furthermore highly dependent upon extracellular Ca^2+^, as it was largely absent in non-recalcified PRP (Fig. 1D-i). This condition also attenuated intracellular Ca^2+^ increases and peak changes within the thrombus (Fig. 1D-ii and iii). To investigate whether synchronised ballooning is associated with [Ca^2+^]_i_ changes within the aggregate, we studied the relationship between peak [Ca^2+^]_i_ and the start of synchronised ballooning. Figure 1E and F demonstrate that synchronised ballooning always followed the peak [Ca^2+^]_i_ of each aggregate, although with some time delay. The data therefore suggest that there is a level of communication within the aggregates that results in synchronised ballooning. As connexin 37 and 40 have previously been shown to regulate thrombosis by allowing communication between platelets in close contact^31–33^, we investigated whether connexin-mediated communication underlie the synchrony of platelet ballooning. The connexin inhibitors Cx40-Gap27 and Cx37-Gap27 however did not alter peak [Ca^2+^]_i_ (Fig. 1G and later 4A) or synchronised ballooning (Fig. 1H).

### Initial central phosphatidylserine exposure and ballooning

**Figure 2:**
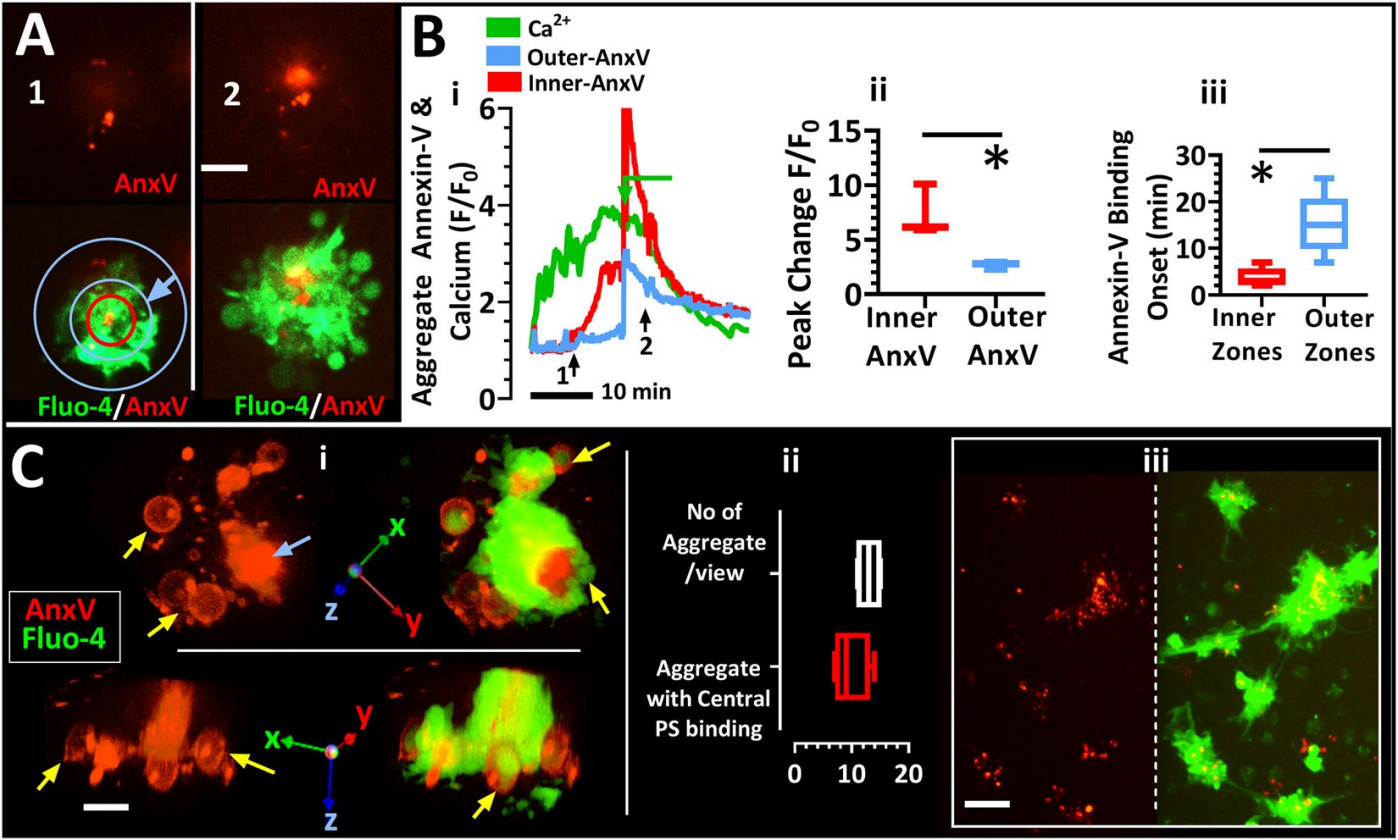
Initiation of Ballooning and Phosphatidylserine Exposure at Central Regions of Aggregated Human Platelets on Collagen-Coated Surfaces. Images are of Alexa568-Annexin-V (AnxV, red) or merged with Fluo-4 (green). (**A**) An aggregate of human platelets in re-calcified platelet rich plasma showing the presence of centralised phosphatidylserine (PS) exposure. Time points of images in A correspond to times indicated by black arrows in Fig B-i. The outer region of the aggregate was defined as ≤6 µm (inwards, blue arrow, A) from the outermost membrane of the aggregate after ballooning and inner region as the circular area covered by 3 µm radius drawn from the point of initial annexin-V accumulation within the aggregate (A). (**B-i**) real-time binding of annexin-V in the outer (between blue circles) and inner (within red circle) zones of the aggregate in (A). Peak change and onset of annexin-V binding in these regions are plotted in B-ii and B-iii, respectively. (**C**) C-i, 3D images of a representative platelet aggregate with the blue arrow pointing to the region of centralised ballooning and PS exposure and the yellow arrows pointing to ballooned platelets. The statistics of this observation are shown in C-ii. C-iii shows a field of view of several aggregates with central PS binding prior to ballooning of platelets in the aggregate periphery. Figure A is related to Movie_S1. Charts B-ii, B-iii and C-ii show interleaved box plots with whiskers showing minimum to maximum values, median, and interquartile range. Data analysis was by Wilcoxon Signed Rank Test. *P < 0.05 was considered significant. Scale bar represents 5 µm (A and C). Data were from 10–15 aggregates (of 7–40 platelets) per view; and 35 ± 5 aggregates were analysed for each of 5 donor blood samples.

To investigate whether ballooning starts in the central or lower regions of the aggregate which correlates to the thrombus core in previous *in vivo* experiments^5, 17^, we imaged aggregate formation in the presence of annexin-V, which binds any externalised PS^34^. In Figure 2A,2Bi, PS data from the aggregate shown in 1B is used to illustrate the simultaneous spatiotemporal exposure of PS in the central region of the growing aggregate. The associated Movie S1 revealed that the first few platelets to make contact with the collagen coated surface rapidly exposed PS likely in tandem with membrane ballooning as we have previously observed^17^. Platelets were thereafter consecutively recruited to this site (Fig. 2A, Movie S1). The region of annexin-V binding remained at the aggregate core until ballooning was observed in the aggregate periphery (Fig. 2A). We determined the temporal externalisation of PS at aggregate core and periphery using Volocity software. We defined the outer region of the aggregate as ≤6 µm (inwards, blue arrow Fig. 2A) from the outermost membrane of the aggregate after ballooning and inner region as the circular area covered by 3 µm radius drawn from the point of initial annexin-V accumulation within the aggregate (Fig. 2A). The results displayed in Fig. 2B-i and B-ii confirmed initial PS exposure in the aggregate centre, with a significant change in peak intensity in the inner region (Fig. 2Bii) and subsequent externalisation of PS at the aggregate periphery (outer region) after a significant delay (Fig. 2B-iii). We assessed this phenomenon in several aggregates from different donors to establish its *in vitro* occurrence (Fig. 2C).

### Thrombin mediates synchronised membrane ballooning

**Figure 3:**
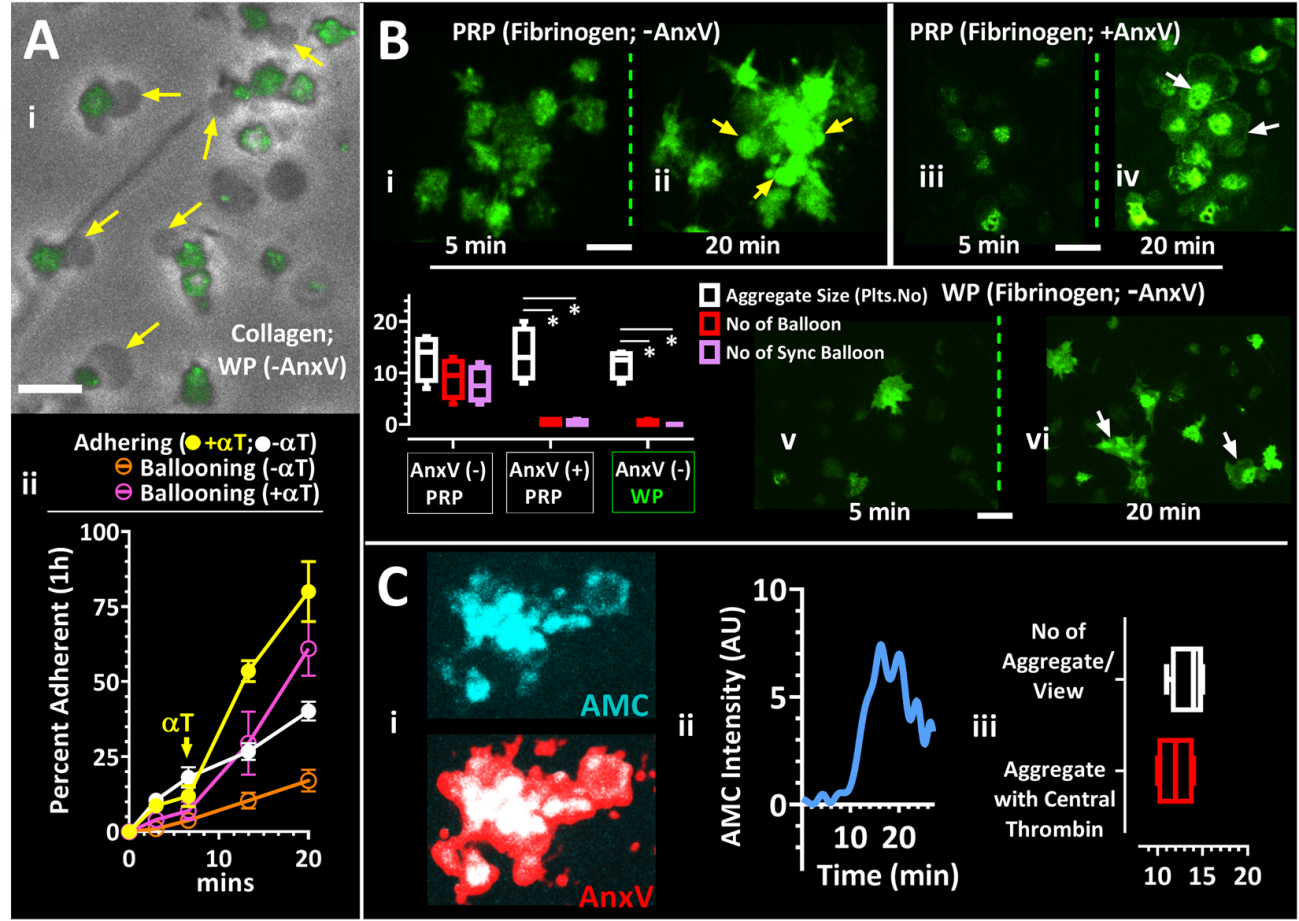
Thrombin mediates synchronised membrane ballooning in aggregating human platelets. (**A**,**B**) (**A**) Fluo-4-labelled washed platelets were allowed to adhere to collagen matrix in the presence of 1 mM extracellular calcium. Yellow arrows in A-i point to platelets induced to balloon by addition of thrombin. A-ii: time course of the percentage of adherent platelets that undergo ballooning, either with addition of α-thrombin (indicated as arrow with αT) or without thrombin addition. Related to Movie_S2. (**B**) Aggregate formation (defined as between 7–40 Fluo-4-labelled platelets in contact with each other) on a fibrinogen-coated surface in Rc-PRP (i-iv) or washed platelets (WP; v-vi). Platelets were pre-incubated with high concentration annexin-V (2%) in B-iii and B-iv. Yellow arrows in B-ii show ballooning platelet membranes. White arrows in B-iv and B-vi indicate lamellipodial spreading. Related to Movies_S3 and S4. The chart in B shows the number of platelets in aggregates, number of total balloons formed per aggregate and the number of balloons formed in a synchronised manner per aggregate. (**C**) C-i, shows extended focus image of fluorogenic thrombin substrate Z-Gly-Gly-Arg aminomethyl coumarin (ZGGR-AMC (cyan) alone and superimposed onto AnxV-stained platelet aggregate (red). C-ii, Fluorescence intensity vs time plot for fluorogenic thrombin substrate. C-iii, shows chart of number of aggregates compared with aggregates showing central thrombin generation per microscope view. Chart in B and Ciii show interleaved box plots with whiskers showing minimum to maximum values, median, and interquartile range. Data analysis was by Wilcoxon Signed Rank Test. *P < 0.05 was considered significant. Scale bar represents 5 µm (A,B,C). Data were from 10–15 aggregates (of 7–40 platelets) per view; and 35 ± 5 aggregates were analysed for each of 5 donor blood samples.

We hypothesised that the mechanism for synchrony may involve release of a soluble agonist. Blocking phosphatidylserine, thrombin or the PAR1 receptor using lactadherin or Annexin-V, PPACK and RWJ-56110 respectively, reduced the peak [Ca^2+^]_i_ increase (Fig. 1G) and substantially diminished synchronised ballooning without affecting ballooning itself (Figs 1H and 3), indicating that thrombin is involved in synchronised membrane ballooning. This was further supported by experiments where exogenously applied thrombin induced synchronised ballooning when added to washed platelets adhering to a collagen-coated surface (Fig. 3Ai-ii, Movie S2). Importantly synchronised ballooning was observed in aggregates formed in PRP on both collagen (Fig. 1A–C) and fibrinogen-coated surfaces (Fig. 3Bi, ii). This is in contrast to washed platelets, which do not balloon upon contact with fibrinogen (Fig. 3B,v-vi). Indeed, ballooning on fibrinogen-coated surfaces occurred only under conditions where thrombin can be generated i.e. PRP, no AnxV (Fig. 3Bi-ii, Movie S3), but not under conditions where it cannot be generated, i.e. PRP plus AnxV (Fig. 3Biii-iv, Movie S4) or washed platelets (Fig. 3Bv,vi). There is therefore a distinction between membrane ballooning due to direct contact with collagen (primary ballooning), as previously described^17^, and later ballooning events in multiple platelets/aggregates, which are synchronous and dependent on thrombin generation. Interestingly, we found that PS exposure, detected by using low concentrations (0.2%) of Annexin-V in order to avoid inhibiting synchronised ballooning, was centrally localised in the newly formed thrombi (Fig. 2). These sites also strongly correlated with the spatial location of active thrombin generation within the aggregates as determined by a fluorogenic thrombin substrate (AMC, Fig. 3C). This observation is thus in agreement with both *in vitro* and *in vivo* studies of thrombus formation on collagen showing PS expression and thrombin formation in the centre of the thrombus^35, 36^. Taken together, these results support the hypothesis that centrally localised PS exposure results in the generation of thrombin, which subsequently induces synchronous [Ca^2+^]_i_ signalling and synchronised ballooning through the PAR1 receptor.

### Synchronised membrane ballooning amplifies platelet microvesicle release

**Figure 4:**
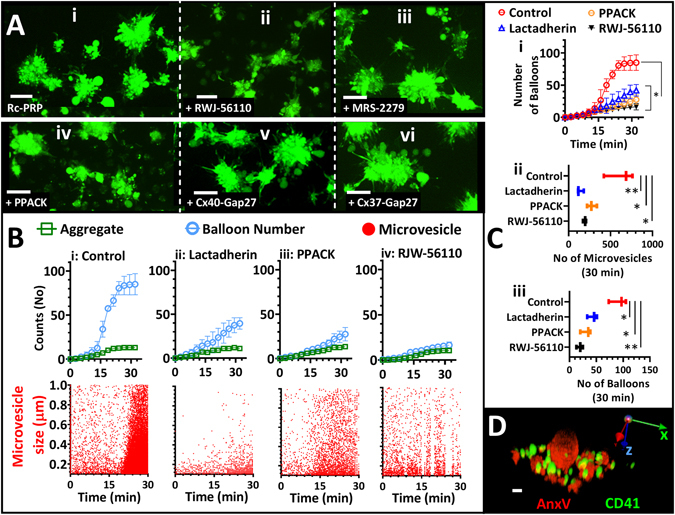
Thrombin-mediated synchronous ballooning is associated with a surge in microvesiculation. (**A**) Aggregate formation of Fluo-4-labelled platelets in re-calcified platelet-rich-plasma (Rc-PRP) in the absence (A-i) or presence of RWJ-56110 (A-ii), MRS-2279 (A-iii), PPACK (A-iv), Connexin40 inhibitor (A-v) or Connexin37 inhibitor (A-vi). Images shown at 1 hr. (**B**) Platelets in Rc-PRP adhering to a collagen-coated surface were monitored for microvesicle release in real time by image-based particle counting. Microvesicles were AnnexinV + and between 100 nm and 1 µm diameter. Display shows scatter plot in the absence (i) or presence of lactadherin (1% ^v^/_v_, ii), PPACK (100 µM, iii) and RWJ-56110 (50 µM, iv). Upper panel B shows corresponding charts of the real time formation of aggregates and the appearance of balloons in the field of view. (**C**) C-i, shows the pooled plot of balloon formation against time data in B (upper panel). C-ii and C-iii show total number of microvesicles (C-ii) and number of balloons (C-iii) at endpoint (30 min) plotted against treatment. (**D**) 3D image of platelet-derived microvesicles stained with Alexa488-conjugated anti-CD41 antibody (green) and Alexa568-Annexin-V (red) show that platelets in Rc-PRP release a mixed microvesicle population. Data analysis was by Friedman test, followed by Dunn’s multiple comparison test. *P < 0.0 5 was considered significant. Scale bar represents 5 µm (A) and 1 µm (D). Cii and Ciii show interleaved box plots with whiskers showing minimum to maximum values, median, and interquartile range. Data were from 10–15 aggregates (of 7–40 platelets) per view; and 35 ± 5 aggregates were analysed for each of 5 donor blood samples.

**Figure 5:**
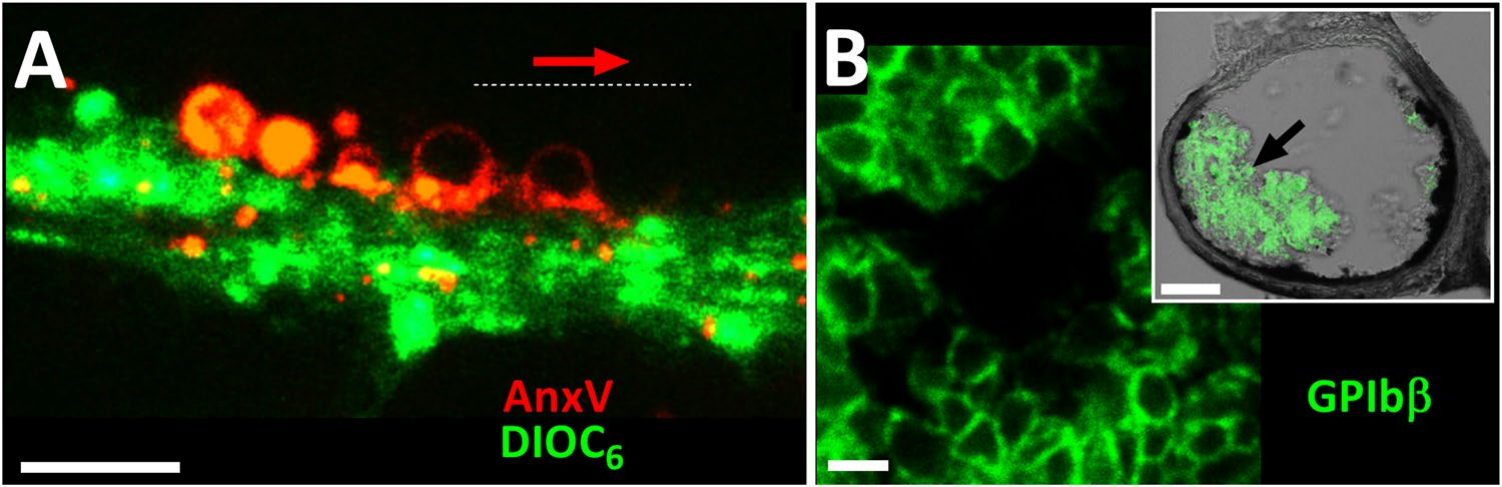
Ballooned platelets are seen after *in vitro* blood flow over collagen and at wound site. (**A**) Whole human blood stained with DIOC_6_ (green) and flowed at 1000s-^1^ over collagen (50 µg/ml) coated surface show ballooned platelets exposing PS (Alexa568-Annexin-V, red) after flow. Red arrow indicates the direction of blood flow. (**B**) Thrombus formed after ferric chloride injury of mouse carotid artery. Platelets were labelled with 488 Dylight conjugated anti-GPIbβ antibody (green) and visualized by super-resolution microscopy (Stimulated Emission Depletion). Cross-section of carotid artery at point of damage is shown in insert, with fluorescent GPIbβ images overlaid on phase contrast. Magnified image of region indicated by black arrow in insert, demonstrate the appearance of ballooned platelets in the structure of the thrombus. Scale bar represents 5 µm (A,B) and 60 µm (insert on B). Data are representative of 6 independent experiments.

**Figure 6:**
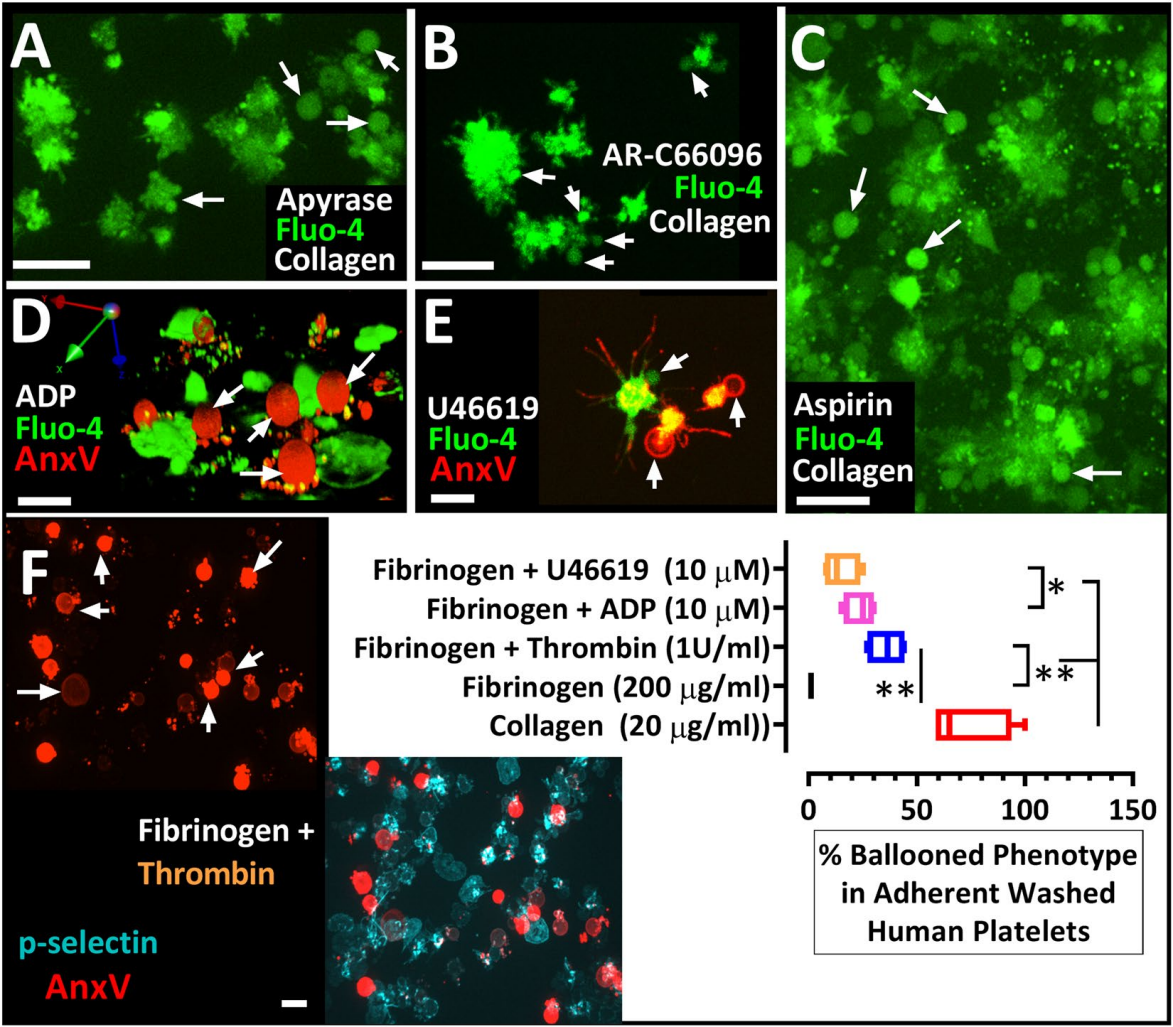
Thrombin-mediated synchronised ballooning is unaffected by ADP and T_X_A_2_ blockade. In (**A**–**C**) platelets in re-calcified platelet-rich-plasma were labelled with calcium dye Fluo-4 and pre-incubated with (**A**) Apyrase (1.5 U/ml), (**B**) AR-C66096 (0.1 µM) and (**C**) Aspirin (10 µM), and were allowed to adhere to a collagen-coated surface. Induction of synchronous membrane ballooning in platelet- rich-plasma is unaffected by ADP and T_X_A_2_ blockade. White arrows point to ballooned platelets in the extended focus images (**A–C**). In (**D–F**), membrane ballooning was induced on fibrinogen-coated surfaces, by ADP, U46619 and thrombin in the presence of 1 mM extracellular calcium. In (**D**,**E** and **F**), 3D (**D**) and extended focus (**E**,**F**) images of AnxV (red) and Fluo-4 (green) or P-selectin (cyan) show ballooned and procoagulant-spread platelets (red) induced by ADP 10 µM (D), U46619 10 µM (E) and 1 U/ml thrombin (F). Images were acquired 1 hr after addition of ADP, U46619 and thrombin and the real-time monitoring of platelet aggregation. Chart in panel F show the percentage of ballooned platelets counted under these condition. Data analysis was by Friedman test, followed by Dunn’s multiple comparison test. *P < 0.0 5 was considered significant. Scale bar represent 5 µm (D,E), and 10 µm (A–C). Data were from 10–15 aggregates (of 7–40 platelets) per view; and 35 ± 5 aggregates were analysed for each of 5 donor blood samples.

The finding that synchronised ballooning occurs after aggregates have formed suggests a role in thrombus consolidation and/or micro-vesicle formation, rather than initial amplification of platelet activation. Indeed, when visualising PRP microvesicle release in real time (Fig. 4A–C), we found that synchronised ballooning was closely followed by a surge in the release of micro-vesicles (Fig. 4B,C), which was prevented by blocking synchronised ballooning using lactadherin (Fig. 4Bii), PPACK (Fig. 4B-iii) or RWJ-56110 (Fig. 4B-iv). However, consistent with our previous finding that ballooning amplifies microvesiculation^17^, we observed a significant decrease in the total number of balloons and microvesicles generated under these conditions (Fig. 4C). Blocking cell-cell communication via Gap junctions did not affect primary or synchronous ballooning and thus total number of ballooned platelets in these experiments (Fig. 4A-v,vi). The released PS positive microvesicles were a mixture of CD41 positive and negative (Fig. 4D), which is in agreement with recent literature^37^. Furthermore, ballooned platelets were observed on the outside of thrombi formed on collagen under flow (Fig. 5A) and in thrombi formed upon FeCl_3_ injury in an *in vivo* mouse model (Fig. 5B). Synchronised platelets’ membrane ballooning was unaffected by hydrolysis of ADP (1.5 U/ml apyrase; Fig. 6A), blocking ADP receptors P2Y_12_ (0.1 µM AR-C66096; Fig. 6B) and P2Y_1_ (10 µM MRS2279 (Fig. 4Aiii)) or blocking TxA_2_ formation (10 µM aspirin) (Fig. 6C), indicating released ADP and TxA_2_ are not involved in synchronised ballooning on a collagen-coated surface. Interestingly, ADP and the thromboxane mimetic (U46619) do have the ability to stimulate ballooning of platelets under certain condition, as shown for washed platelets adhered to a fibrinogen-coated surface (Fig. 6D and E), albeit this was in a non-synchronised manner. Thrombin however remained the most potent stimulator of platelet membrane ballooning under this condition (Fig. 6F).

## Discussion

In this study we set out to add further functional and physiological context to human platelet membrane ballooning^17^, by observing the nature of the ballooning event in platelet aggregates, rather than as individual isolated cells adherent to a collagen matrix as previously reported^17^. In order to do this we chose to observe aggregate formation of platelets added in plasma to the collagen-coated surface. We also focused on smaller aggregates, the number of platelets within which can be confidently quantified, for the statistical evaluation of our findings. Under these conditions, where platelets are bathed in physiological concentrations of fibrinogen and the full complement of coagulation factors, we observed a surprising event. Platelets accumulated on the collagen-coated surface, clumping together in aggregates, and multiple platelets within an aggregate all underwent ballooning approximately simultaneously. This strongly suggested that there was communication between the cells in the aggregate, and we hypothesised that this was the result either of direct physical contact between cells, or due to the release of a soluble mediator that diffused rapidly to stimulate the platelets in the aggregate simultaneously. In this study we addressed the mechanism underlying the synchrony of ballooning, which is mediated by generated and released thrombin. The study also adds further detail to the physiological context of synchronised ballooning, since it acts as a further positive feedback mechanism in platelets to generate a burst of thrombin and also a surge in the release of procoagulant platelet-derived microvesicles.

**Figure 7:**
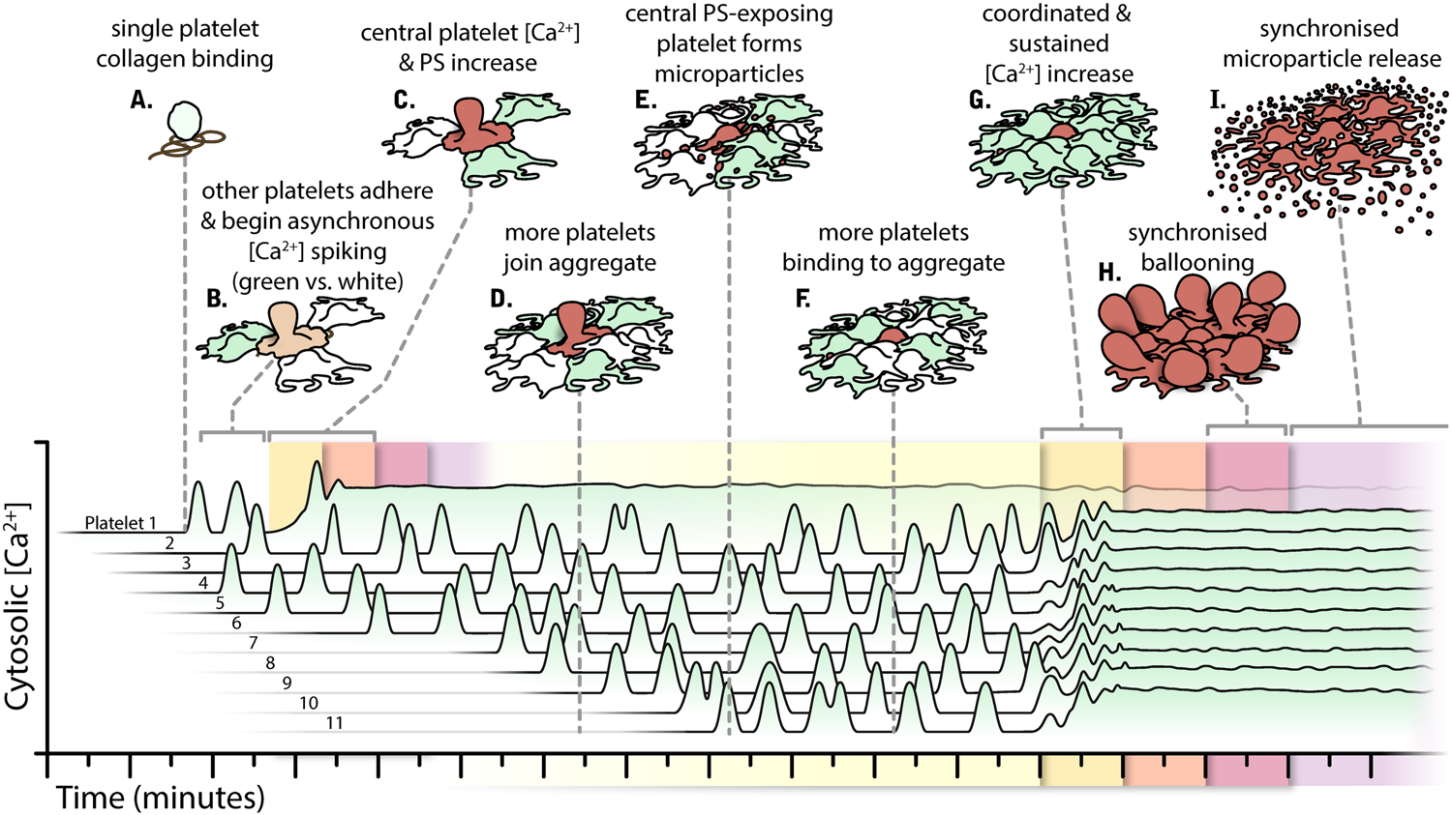
Synchronous membrane ballooning in human platelet-rich plasma *in vitro*. In this model, platelets adhere to collagen upon vessel injury (**A**), and undergo primary procoagulant membrane ballooning (**B**–**D**) without synchronising individual calcium fluxes. These platelets bind the prothrombinase complex to support an initial localised thrombin formation (**E–G**), which diffuses to activate peripheral platelets and induce synchronised secondary membrane ballooning at the outer layer of the thrombus (**H**). Due to the action of thrombin on PAR1 receptors, this dramatic morphological event is preceded by the synchronisation of the cytosolic calcium flux in the individual platelets of the formed aggregate. The result is a subsequent rapid increase in the number of ballooned platelets and a surge in the number of platelet-derived microvesicles (**I**). This leads to a rapidly amplified but coordinated generation of more thrombin to stabilise the haemostatic response through coagulation. The image was conceptualised and outlined by the authors, and then drawn by ScideLight illustrations (www.ScideLight.com).

A key mechanistic observation, when studying the videos of the event closely (Movie S1), was that there is a characteristic pattern to the calcium signalling in the platelets of the aggregate. When platelets join the growing aggregate they start an intermittent but non-coordinated spiking in their intracellular calcium concentration. At a certain time point however the majority of platelets in the aggregate appear to coordinate their cytosolic calcium rise, so that they simultaneously stop spiking and undergo a rise in their intracellular calcium to a sustained plateau. At this point they then undergo synchronised ballooning, as illustrated in Fig. 1B and in the associated video (Movie S1). It is clear that a sustained rise in cell calcium is critical for mediating PS exposure in platelets^38, 39^, and that it plays a key role in mediating ballooning also^14, 17^. The data here support these observations, since it appears that transient calcium spiking is insufficient to induce platelet ballooning, which only occurs after a sustained or synchronised rise in the calcium concentration in the cells (Figs 1D–F and 7).

The observations of synchronised calcium preceding synchronous membrane ballooning also raised the question about the nature of the signal that caused the conversion of spiking events to sustained plateau or synchronised signal, and how that signal was transmitted simultaneously to multiple cells in the aggregate periphery. Thrombin has been shown in this study to be the key mediator of synchronised aggregate ballooning on collagen, since inhibition of thrombin activity or blockade of PAR1 is sufficient to ablate synchrony. This is in contrast to other possible mechanisms of synchrony, which included released ADP or TxA_2_. It is possible that the stimulus for synchrony needs to be a potent stimulus, which would explain why thrombin is able to mediate the response but not weaker agonists such as ADP or TxA_2_. Interestingly, platelets in PRP, but not washed platelets, balloon when adhered to a fibrinogen-coated surface. Under these conditions, ADP and TxA_2_ were able to induce ballooning of washed platelets adhered to fibrinogen, although not to the same extent as thrombin (Fig. 6D–E). Indeed, preventing thrombin formation blocked synchronised aggregate ballooning on fibrinogen in PRP (Fig. 3B). Together, these results suggest that although ADP and TxA_2_ have the potential to contribute to the ballooning response, their effect is likely to be redundant in the setting of a platelet aggregate where thrombin was allowed to be generated.

Alone, thrombin is a known potent inducer of platelet membrane ballooning^8^. In our study, this was also evident in washed platelets conditions where addition of thrombin induced ballooning in fibrinogen-adherent platelets (Fig. 6F). Notably, our data in washed platelets contrasted those previously reported by Heemskerk and coworkers^14^ who showed that only about 1% of platelets adherent to fibrinogen became procoagulant with the addition of thrombin. One reason for this might be the lower concentration of thrombin (2 nM) used in Heemskerk study compared to this study (IU/ml ≈10 nM). Also, there was no blockade of cyclooxygenase in the final imaged washed platelet samples in our experiments, although platelets were treated with indomethacin for the preparation steps, this was excluded in the final resuspension, leaving cyclooxygenase uninhibited. An alternative explanation for synchrony was direct cell-cell communication, through contact, between platelets. Connexins have been recently shown to play roles in mediating such communication^31–33^, but although these clearly have roles to play in aggregate formation, they do not seem to play a role in the synchronised ballooning function. In addition, synchronous membrane ballooning is unlikely to be the product of random ballooning events since some treatments, such as lactadherin and PPACK, abolished synchronous ballooning without having an effect on total ballooning (Fig. 4B). Furthermore, unlike primary ballooning in multiple platelets, synchronous ballooning always followed a sustained or synchronised rise in intracellular calcium (Figs 1D–F and 7).

Moreover, if thrombin mediates synchronised ballooning, which in turn leads to microparticle release and a burst of procoagulant activity generating more thrombin, the process represents a positive feedback loop. There needs therefore to be an initiating or ‘seed’ generation of thrombin, and this is likely to be generated by the platelets that become procoagulant upon direct contact with collagen. Indeed, Fig. 2 shows that this is the case, where platelets in the core of an aggregate, before synchronised ballooning takes place, show exposure of surface PS, and generation of thrombin locally at the centre of the aggregate (Fig. 3C). It is therefore this local thrombin generation that we believe triggers the synchronised wave of balloon formation in the periphery of the aggregate. In turn then, these platelets expose PS, and shed multiple microvesicles, thereby massively amplifying the thrombin that is generated. So, this is effectively thrombin-induced thrombin generation, through platelet ballooning and microvesicle shedding, and is an example of positive feedback signalling in platelet-dependent clot formation. This is likely to be a critical feature of rapid clot formation required in thrombi *in vivo*. Importantly, the surge in microvesicle release is prevented when synchronous ballooning is also prevented (Fig. 4B). This study therefore reveals the potential physiological importance and function of synchronised platelet ballooning as a mechanism for microvesicle formation and amplification of local coagulation. Furthermore, we now demonstrate a complex feed-forward role for thrombin in its own generation, through its regulation of platelet membrane ballooning and microvesicle formation.

We previously reported that ballooned platelets adherent to collagen also generate procoagulant microvesicles^17^ and microvesicle production is thus not dependent on synchronized ballooning. However, this study does reveal a significant surge in microvesicle numbers associated with synchronous ballooning, which is markedly reduced by thrombin inhibition. Previous work suggested an important role for thrombin in the generation of procoagulant microvesicle. Platelets from platelet-specific TMEM16F-deficient mice exhibited defects in activation-induced procoagulant microvesicle generation that are associated with a severely reduced ability to generate thrombin^40^. The synchronicity observed in this study operates, at least *in vitro*, and the surge in the release of microvesicles may be physiologically important to achieve a surge in thrombin generation and the rapid conversion of soluble fibrinogen to fibrin at wound sites. However, the ‘spreading’ of calcium signal from one platelet to another is also important, and the sustained Ca^2+^ rise in the aggregate may have amplified the total response required for synchronous ballooning. Indeed, a key finding of our study is that extracellular calcium is required for synchronised ballooning. However, a direct role for sustained aggregate Ca^2+^ in synchronised ballooning is difficult to confirm as extracellular Ca^2+^ is essential for primary ballooning^17^, without which initial thrombin generation and subsequent synchronised ballooning will not occur.

By not having a flow environment, we expect that thrombin and microvesicles generated are not rapidly washed away. This allows us to easily detect ballooning, thrombin and microvesicle generation under these conditions. We expect that when flow is applied, the thrombin generated in the microenvironment on the inside of the thrombus will be in a protected environment and able to diffuse to the outside of the thrombus and induce ballooning, thrombin and microvesicle generation. This is consistent with reports from systems approaches which have combined *in vitro*, computational and *in vivo* models to understand the regulation of PS exposure and the actions of soluble agonists in space and time^41–44^. These studies demonstrate the feasibility of centrally generated thrombin from highly activated platelets and the containment of this agonist at high concentrations within the central thrombus core due to reduced porosity and increased cell packing in this region^41–44^.

A limitation of the current study is the lack of a clear demonstration, *in vivo*, of a role for synchronized membrane ballooning. This is because there is no inhibitor that is able to block synchronized membrane ballooning without also either blocking membrane ballooning per se or blocking thrombin activity, leading to an anticoagulant action, which would mask the haemostatic or thrombotic effects of synchronized membrane ballooning. For example, the fluorogenic thrombin substrate Z-Gly-Gly-Arg-aminomethyl coumarin (Z-GGR-AMC) like other thrombin substrates enable the visualisation and spatial localisation of thrombin generation; however these substrates release the fluorescent AMC which is measured, only after cleavage by thrombin. The substrate continues to occupy the active sites of thrombin, thereby inhibiting its activity but prolonging its lifetime^45, 46^. Accordingly, we did not expect active thrombin to accumulate in experiments where we determined thrombin generation and indeed we did not observe SMB under this condition. However, our data revealed the spatial localisation of thrombin generation, particularly at sites which correlated to regions of initial central PS exposure. This notwithstanding, our study strongly suggests that ballooning synchrony is likely to be a mechanism by which rapid fibrin generation can be achieved at the site of tissue damage, whilst also maintaining localisation of the thrombus. This work therefore provides a testable hypothesis for future *in vivo* work.

In conclusion therefore, based upon our findings and previous literature, we propose a model (Fig. 7) where upon vessel injury, platelets adhere to collagen and undergo primary procoagulant ballooning. These become responsible for an initial localised thrombin formation resulting in synchronised secondary membrane ballooning in platelets at the outer layer of the thrombus. This then leads to a subsequent surge in the release of platelet-derived microvesicles, and to a massively amplified but coordinated generation of thrombin to stabilise the haemostatic response through coagulation.

## Experimental Procedures

### Consent of Human Blood Donors and Methods

Written informed consent was obtained in accordance with the Declaration of Helsinki. Human blood was obtained from healthy drug-free volunteers under the University of Bristol, United Kingdom, Research Ethics approval (E5736). All methods were performed in accordance with the University of Bristol research guidelines and regulations.

### Animals

Male C57BL/6 mice aged 8–10 weeks (20–25 g) were used for all animal experiments. Mice were bred and maintained in the University of Bristol animal facility in accordance with United Kingdom Home Office regulations. All procedures were undertaken with United Kingdom Home Office approval in accordance with the Animals (Scientific Procedures) Act of 1986 (project license number 30/2908).

### Materials

Fibrillar collagen (Horm suspension) was from Nycomed (Munich, Germany). Alexa Fluor® 568 conjugated annexin V (annexin-V; anxV) and Fluo-4 AM, were purchased from Life Technologies. Alexa Fluor® 568-Labelled Lactadherin was purchased from Cambridge Bioscience Ltd. Bovine serum albumin (BSA), ADP, Aspirin and Apyrase were from Sigma Aldrich. RWJ-56110, MRS 2279 and AR-C66096 were from Tocris (Bristol, UK). PPACK was from Enzo life science. Connexin inhibitors, Cx37Gap27 (SRPTEKTIFII) was from Sigma Aldrich and Cx40Gap27 (SRPTEKNVFIV) was from Anaspec, USA. Glass bottom 35 mm dishes (P35G-1.5-20-C) were obtained from MatTek Corporation (USA). Flow chambers (Vena8^TM^ biochip) were from Cellix Ltd. The fluorogenic thrombin substrate Z-Gly-Gly-Arg aminomethyl coumarin (Z-GGR-AMC) was from Bachem AG. Dylight 488–conjugated anti-GPIbβ antibody was from Emfret Analytics, Eibelstadt, Germany.

### Human Platelet Rich Plasma and Washed Platelet Preparation

Blood drawn from healthy human volunteers was anticoagulated with 0.4% trisodium citrate and centrifuged at 180 g for 17 min, to prepare platelet-rich plasma (PRP). To prepare washed platelets, PRP was centrifuged at 650 *g* for 10 minutes in the presence of 10 μmol/L indomethacin and 0.02 U/mL apyrase, and pellet resuspended in HEPES-Tyrode buffer modified with 0.1% (wt/vol) glucose.

### Live Platelet Confocal Microscopy

Platelets were pre-incubated (10 mins) with calcium dye Fluo-4 AM with or without other probes as indicated in figures and legends. In most experiments, PRP was re-calcified with 4.5 mM CaCl_2_ (Rc-PRP). Fibrillar collagen (20 μg/mL) or fibrinogen (200 µg/ml) were used to pre-coat MatTek dishes and then blocked with 2% fatty acid-free BSA. Aliquots of PRP were added onto dishes and platelet activities were monitored in xy or xyz dimensions over time. High resolution 3D and 4D images of live-platelets aggregating over agonist coated surfaces, in platete-rich-plasma were obtained at 25 °C using spinning-disk confocal system as previously described^17^. In washed platelet experiments, platelets were suspended in Tyrode’s supplemented with 1 mmol/L CaCl_2_.

### Image Deconvolution and Analysis

Image resolution was improved by the restoration complement of Volocity 6.3 imaging software (Perkin-Elmer, UK). Point-spread functions specific to the microscope and acquisition lenses were calculated using the action menu of the software, this was then used to conduct iterative restoration of the fluorescent images. In this study, we report findings from aggregates of 7–40 human platelets, monitored 10–15 aggregates per microscope view and analysed data from 35 ± 5 aggregates for each donor blood sample. Platelets were judged to aggregate when cell-cell contact was achieved and sustained throughout the period of visualisation. Membrane ballooning in aggregating platelets was deemed to have been synchronised, if preceded by a synchronisation of the calcium fluxes of the aggregating platelets and when ≥60% of balloons formed by these platelets appeared within a 5 minute period. Image analyses were based on these criteria. To determine aggregate cytosolic calcium fluxes, or the regional binding of annexin-V, regions of interest were drawn over extended-focus images of each platelet aggregate. Relative fluorescence intensity (*F*/*F*0) over time was reported, where *F*0 is the background-subtracted fluorescence intensity before platelet activation. Using pre-installed Volocity algorithms, platelet-derived microvesicles were identified by intensity in 4D data using both Fluo-4 and Annexin-V signal intensities combined with a size discrimination step for vesicles of size between 100 nm and 1 µm, which excluded vesicles <100 nm and >1 µm from the analysis output. Image rotations and movie creation from 3D & 4D data as well as the computation of percentage co-occurrence of signal intensities were performed using Volocity 6.3 imaging software (Perkin-Elmer, UK).

### Stimulated Emission Depletion (STED) Microscopy

STED imaging of samples was achieved through use of 592 nm (for Alexa 488 excited at 488 nm) and 660 nm (for Alexa 568 excited at 561 nm) depletion lasers using the Leica STED 3X system (Leica Microsystems). Fluorescence was collected in the same bandwidths using time gated hybrid detectors. Time gating was set to 1.8–6.5 ns for both Alexa 488 and Alexa 568 detection to ensure complete depletion of fluorescence within the STED doughnut area. Notch filters at 488, 561, 592 and 660 nm were used to suppress any stray laser light. Images of Alexa 568 were acquired first to avoid bleaching by the 592 nm depletion laser used for Alexa 488.

### *In vitro* thrombosis assay

Flow chambers (Vena8^TM^ biochip) were precoated with 20 μg/mL fibrillar collagen overnight at 4 °C and then blocked for 2 h at room temperatre with 2% fatty acid-free BSA (Sigma Aldrich). Blood was drawn into solutions of 4% citrate (1:10 v/v) and then labelled with 2 μmol/L DiOC_6_ (3,3′-dihexyloxacarbocyanine iodide) for 10 min in the dark. Blood samples were recalcified prior to flow through flow chambers with 6.6 mmol/L CaCl_2_ and 6.6 mmol/L MgCl_2_. Blood was then made to flow through the chambers at a rate of 1000 s^−1^ for 5 min. The flow chambers were then washed in quick succession with solutions of Alexa568-Annexin-V (1% v/v) and then with HEPES-Tyrode’s buffer (10 mmol/L HEPES pH 7.2, 145 mmol/L NaCl, 3 mmol/L KCl, 0.5 mmol/L Na_2_HPO_4_, 1 mmol/L MgSO_4_) for 5 min to remove excess red blood cells. The chambers were then visualised within 2 h, on a spinning-disk confocal microscope.

### Measurement of thrombin generation in platelet aggregates

Thrombin generation was initiated by the addition of 5 pmol/L tissue factor to re-calcified platelet-rich-plasma pre-incubated with fluorogenic thrombin substrate Z-Gly-Gly-Arg aminomethyl coumarin (Z-GGR-AMC; 450 µmol/L). Fluorescence intensities of Z-GGR-AMC was measured in whole platelet aggregates or at regions of interest. The ensuing data were converted into first-derivative curves.

### Ferric chloride carotid injury model in mouse

*In*-*vivo* experiments were carried out as previously described^17^. Briefly, mice were anaesthetised with a ketamine-xylazine mix (100 mg/kg and 10 mg/kg respectively). Platelets were labelled with Dylight 488-conjugated anti-GPIbβ antibody and carotid arteries were exposed to 12% ferric chloride for 3 minutes prior to observation by fluorescence time-lapse microscopy.

### Data analysis and statistics

Data were analysed using GraphPad Prism 7 (San Diego, CA) and presented as box with whiskers plots showing min to max values, the medians and interquartile ranges of data. Test for normality was by D’Agostino & Pearson omnibus as well as Shapiro-Wilk tests. Statistical significance was determined by the Friedman test, followed by Dunn’s multiple comparison test or by Wilcoxon Signed Rank Test. *p < 0.05 or **p < 0.01 was considered significant.

## Electronic supplementary material

Movie S1

Movie S2

Movie S3

Movie S4
